# Adrenal Crisis: From Perioperative Clinic to Diagnosis

**DOI:** 10.7759/cureus.88971

**Published:** 2025-07-29

**Authors:** Ahmet Gültekin, Kağan Berkay Öner, İlker Yıldırım, Cavidan Arar, Selami Gürkan

**Affiliations:** 1 Department of Anesthesiology and Reanimation, Tekirdağ Namık Kemal University Faculty of Medicine, Tekirdağ, TUR; 2 Department of Cardiovascular Surgery, Tekirdağ Namık Kemal University Faculty of Medicine, Tekirdağ, TUR

**Keywords:** adrenal crisis, adrenal insufficiency (ai), high-dose vasopressors, perioperative hypotension, persistent electrolyte imbalances

## Abstract

Adrenal crisis, a life-threatening complication of adrenal insufficiency, can present perioperatively with refractory hypotension and electrolyte imbalances. This case report describes a 77-year-old woman with multiple comorbidities who developed intraoperative shock unresponsive to high-dose vasopressors during aortobifemoral bypass surgery. Persistent hypokalemia and hypoglycemia raised suspicion of adrenal insufficiency, prompting empiric intravenous (IV) methylprednisolone (75 mg), which rapidly resolved hypotension and obviated further inotropic support. The case underscores the importance of considering adrenal crisis in perioperative refractory hypotension, even without prior diagnosis. Early glucocorticoid administration, prior to confirmatory laboratory results, is critical to prevent mortality.

## Introduction

Adrenal crisis, or Addisonian crisis, is a life-threatening endocrine emergency caused by acute adrenal insufficiency, typically due to inadequate cortisol production. This may result from primary adrenal failure, the suppression of the hypothalamic-pituitary-adrenal (HPA) axis, or triggers such as infection, trauma, or abrupt steroid withdrawal. Prompt recognition and treatment are essential, as delays can lead to fatal outcomes [1]. Clinicians must distinguish adrenal crisis from adrenal insufficiency, as the former requires urgent intervention. According to a 2019 study by Rushworth et al., adrenal crisis is marked by acute clinical deterioration with either absolute hypotension (systolic blood pressure {SBP}: <100 mmHg) or relative hypotension (≥20 mmHg drop from baseline) [2]. These signs typically improve within 1-2 hours of parenteral glucocorticoid administration, with hypotension often resolving within the first hour. Further evaluation is necessary to identify underlying causes and secondary triggers. In patients without known adrenal disease presenting with hypotension resistant to fluids and vasopressors, adrenal crisis should be strongly suspected [2].

Differential diagnoses include the following:* *shock: cardiogenic, obstructive, distributive, hypovolemic, and septic; cardiac: acute myocardial infarction; endocrine: thyrotoxicosis, diabetic ketoacidosis, hyperosmolar state, myxedema coma, and pituitary disorders; GI causes: dehydration, gastroenteritis, poor intake, and acute abdomen; hematology and oncology: malignancy or immunotherapy history; obstetric: hyperemesis gravidarum; infectious: localized/systemic infections; and stressors: recent surgery, trauma, and psychological stress [1].

Adrenal insufficiency presents with a wide range of symptoms, from mild and nonspecific to life-threatening shock. Because its symptoms are often vague and its clinical presentation varies, clinicians must maintain a high index of suspicion for this condition. Diminished or suppressed adrenal function can remain hidden until stress or illness triggers an adrenal crisis [3]. It is well known that endogenous cortisol secretion increases significantly in healthy individuals in response to stressful events. In contrast, patients with adrenal insufficiency need to adjust their glucocorticoid doses appropriately in the event of stress to meet the increased demand for adrenal steroids and prevent adrenal crisis [4]. Adrenal crisis may be the first manifestation in a previously undiagnosed patient in whom an acute major stressor appears to tip the balance toward overt hypotension [5]. Patients with known primary or secondary adrenal insufficiency require supplemental glucocorticoids to prevent adrenal crisis in the perioperative setting. There are no good data regarding the development of perioperative hypotension and the need for rescue corticosteroids and fluids among those patients not treated with stress-dose corticosteroids [6].

## Case presentation

This case report describes a 77-year-old woman with diabetes mellitus (insulin-dependent type 1 diabetes), hypertension, chronic renal failure, and coronary artery disease. She has a history of a coronary artery bypass graft operation six years ago. Following a cardiology consultation, the patient's ejection fraction was found to be normal, with no signs of cardiac tamponade. The patient was planned for an aortobifemoral bypass procedure. During the induction of general anesthesia, 90 mg propofol, 40 mg rocuronium, and 40 mcg fentanyl were administered to the 45 kg patient. Following endotracheal intubation, the patient was monitored using invasive arterial monitoring. Two additional doses of a neuromuscular blocker were administered during the approximately four-hour operation. In addition to fluid therapy, two units of erythrocyte suspension and electrolyte replacement were administered for blood gas monitoring during the last two hours of the surgery. In addition to the medical treatments used for her chronic diseases, the patient also received replacement treatments for hyponatremia and hypokalemia throughout her hospital treatment.

However, 20 minutes after the onset of the condition, the patient required an inotrope, and dopamine and then dobutamine infusions were initiated, increasing the dose to the maximum (20 mcg/kg/minute). Approximately 20 minutes after the start of inotropes, noradrenaline infusion was started due to deepening hypotension, and the maximum infusion dose (1 mcg/kg/minute) was reached within 20 minutes. During the patient's hospitalization and follow-up, hypokalemia, electrolyte imbalances, and hypotension that did not respond to perioperative high-dose vasoactive drugs were observed, and it was thought that the patient may have an inadequate adrenal response to surgical stress. After perioperative intravenous (IV) 75 mg methylprednisolone (because it is the fastest and easiest steroid we can access) was administered to the patient, the patient's norepinephrine requirement was finished within a few minutes, and inotrope infusions were turned off after 10 minutes to make the patient normotensive. The patient was transferred to the intensive care unit in an intubated, normoglycemic, normotensive state without inotropic support.

## Discussion

Adrenal insufficiency may present with nonspecific symptoms (e.g., fatigue, weight loss, nausea, and anorexia), potentially causing a delay in diagnosis. In a cross-sectional study of 216 patients with both primary and secondary adrenal insufficiency, 47% had symptoms for more than one year before diagnosis, and 20% had symptoms for more than five years before diagnosis [7]. Adrenal insufficiency is a rare disease, and therefore, many doctors are unfamiliar with its management, often resulting in critical delays in emergency treatment [8].

This case highlights adrenal crisis as a reversible yet frequently overlooked cause of perioperative cardiovascular collapse. The patient's abrupt response to glucocorticoids strongly supported adrenal insufficiency as the underlying etiology, consistent with the pathophysiology of inadequate cortisol secretion during stress [3]. Notably, hypokalemia, an atypical feature for primary adrenal insufficiency, suggested secondary insufficiency, possibly due to chronic glucocorticoid use or pituitary dysfunction [9]. This supports the idea that our case had a delayed diagnosis.

## Conclusions

In patients with persistent electrolyte imbalance and hypotension, adrenal crisis should be considered in the preoperative evaluation. In hypotension unresponsive to high-dose inotropic drugs perioperatively, adrenal insufficiency or crisis should be considered among the diagnoses, and drugs should be added to the treatment accordingly. In case of hypokalemia, as in this case, the cause of adrenal insufficiency may be secondary and should not be overlooked. Before starting steroid treatment, blood should be taken for serum cortisol, renin, adrenocorticotropic hormone (ACTH), and biochemical tests. However, treatment should not be delayed while waiting for blood test results.
